# Benznidazole

**DOI:** 10.1107/S1600536808005023

**Published:** 2008-02-27

**Authors:** José Lamartine Soares-Sobrinho, Marcílio S. S. Cunha-Filho, Pedro José Rolim Neto, Juan J. Torres-Labandeira, Bruno Dacunha-Marinho

**Affiliations:** a Programa de Pós-graduação em Ciências Farmacêuticas, Universidade Federal de Pernambuco, 50740-521 Recife, Brazil; b Departamento de Farmacia y Tecnología Farmacéutica, Facultad de Farmacia, Universidad de Santiago de Compostela, 15872, Spain; c Ed. CACTUS, Campus Sur, Unidade de Raios X, Universidad de Santiago de Compostela, 15782, Spain

## Abstract

The conformation of the title compound [systematic name: *N*-benzyl-2-(2-nitro­imidazol-1-yl)acetamide], C_12_H_12_N_4_O_3_, can be described in terms of the relative orientation of three planar fragments,  the imidazol group (*A*), benzyl group (*B*), and the acetamide fragment (*C*), with corresponding dihedral angles: *A*/*C* = 88.17 (4), *B*/*C* = 67.12 (5) and *A*/*B* = 21.11 (4)°. The crystal packing is enhanced by a network of strong inter­molecular N—H⋯O hydrogen bonds.

## Related literature

For related literature, see: Coura & Castro (2002 ▶); Lamas *et al.* (2006 ▶); Morilla *et al.* (2004 ▶); Silva *et al.* (2007 ▶).
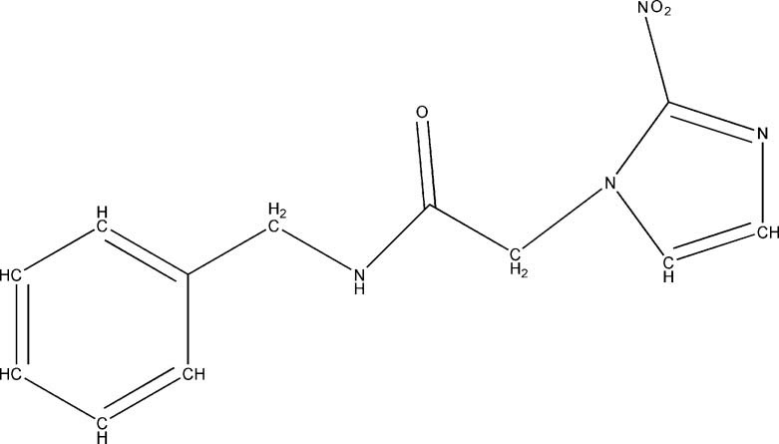


## Experimental

### 

#### Crystal data

C_12_H_12_N_4_O_3_
*M* *_r_* = 260.26
Monoclinic, 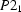

*a* = 4.65560 (10) Å
*b* = 10.9113 (2) Å
*c* = 11.7681 (3) Å
β = 90.6680 (10)°
*V* = 597.76 (2) Å^3^
*Z* = 2
Mo *K*α radiation
μ = 0.11 mm^−1^
*T* = 100 (2) K
0.34 × 0.16 × 0.12 mm

#### Data collection

Bruker APEXII CCD diffractometer
Absorption correction: multi-scan (*SADABS*; Bruker, 2001 ▶) *T*_min_ = 0.861, *T*_max_ = 0.987
11597 measured reflections
1287 independent reflections
1216 reflections with *I* > 2σ(*I*)
*R*_int_ = 0.012

#### Refinement

*R*[*F* ^2^ > 2σ(*F* ^2^)] = 0.025
*wR*(*F* ^2^) = 0.066
*S* = 1.08
1287 reflections
176 parameters
1 restraint
H atoms treated by a mixture of independent and constrained refinement
Δρ_max_ = 0.14 e Å^−3^
Δρ_min_ = −0.22 e Å^−3^

### 

Data collection: *APEX2* (Bruker, 2007 ▶); cell refinement: *APEX2*; data reduction: *APEX2*; program(s) used to solve structure: *SIR97* (Altomare *et al.*, 1999 ▶); program(s) used to refine structure: *SHELXL97* (Sheldrick, 2008 ▶); molecular graphics: *Mercury* (Macrae *et al.*, 2006 ▶) and *ORTEP-3 for Windows* (Farrugia, 1997 ▶); software used to prepare material for publication: *WinGX* (Farrugia, 1999 ▶) and *PLATON* (Spek, 2003 ▶).

## Supplementary Material

Crystal structure: contains datablocks I, global. DOI: 10.1107/S1600536808005023/om2213sup1.cif

Structure factors: contains datablocks I. DOI: 10.1107/S1600536808005023/om2213Isup2.hkl

Additional supplementary materials:  crystallographic information; 3D view; checkCIF report

**Fig. 3.**
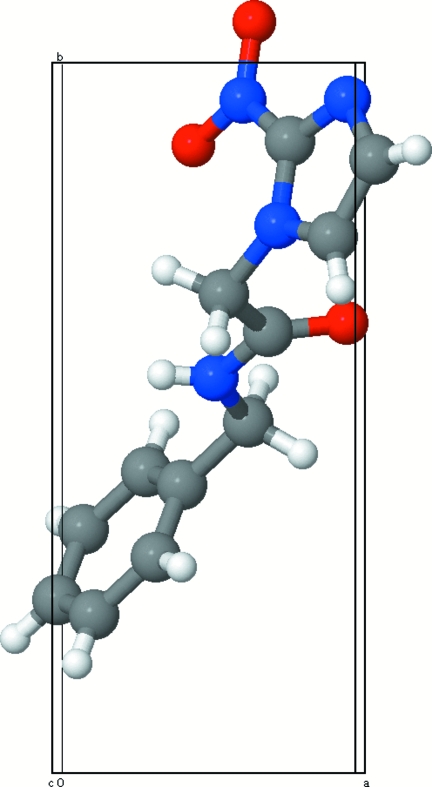
An interactive view of Benznidazole.

Enhanced figure: interactive version of Fig. 3

## Figures and Tables

**Table 1:** Hydrogen-bond geometry (Å, °)

*D*—H⋯*A*	*D*—H	H⋯*A*	*D*⋯*A*	*D*—H⋯*A*
N8—H8⋯O10^i^	0.835 (19)	2.037 (18)	2.837 (2)	160.2 (17)

Symmetry code: (i) 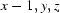.

**Fig. 1.**
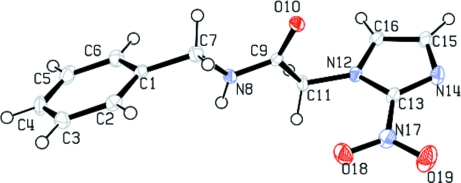
The molecule of Benznidazole showing the atom-labeling scheme. Displacement ellipsoids are drawn at the 50% probability level.

**Fig. 2.**
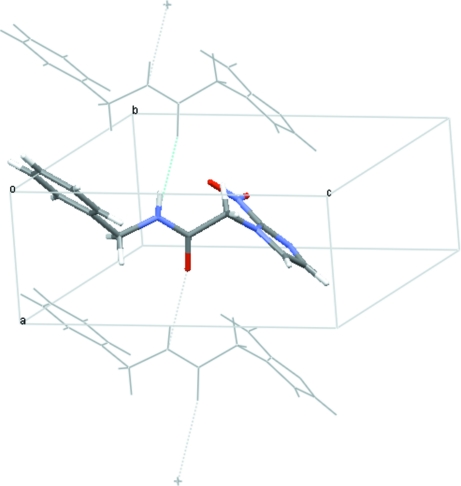
Hydrogen bond network along the a direction.

## supplementary crystallographic information

### Comment

The title compound, Benznidazole, is the drug of choice in the treatment of Chagas disease, a protozoan infection caused by the parasite *Trypanosoma cruzi*. This illness constitutes a major public health problem for developing nations, affecting sundries Latin America countries, being considered a neglected disease according to World Health Organization (Coura & Castro, 2002; Lamas *et al.*, 2006). In spite of the epidemiological importance of this disease, currently the only available therapeutic agent is Benznidazole, especially effective in the acute phase of infection (Morilla *et al.*, 2004).

The conformation of the title compound (Fig. 1) can be described by the mutual orientation of three approximately planar fragments, A, B and C: one imidazole group (fragment A: N12, C13, N14, C15, C16, N17, O18 and O19 atoms) for which the maximum deviation from the least-squares plane is -0.067 (1) Å, the benzene group (fragment B: C1, C2, C3, C4, C5, C6 and C7 atoms) whose maximum deviation from the mentioned planarity is -0.013 (2), and the central acetamide (fragment C: N8, C9, O10 and C11 atoms), with a deviation of 0.024 (1) Å. The corresponding dihedral angles are: A/C = 88.17 (4)°, B/C = 67.12 (5)° and A/B = 21.11 (4)°.

The strategy of self-assembly through weak interactions is of central importance for efficient and specific biological reactions. In our case we can find one strong intermolecular O···HN hydrogen bond between N(8)–H(8) and O(10) that almost lies along the "a" axis (Fig. 2).

### Experimental

Benznidazole was supplied by Laboratório Farmacêutico do Estado de Pernambuco/LAFEPE batch 13871(Recife, Brazil). Purity was estimated by differential scanning calorimetry (DSC-50 Shimadzu) and high-performance liquid chromatography (HPLC Shimadzu) and found to be 99.9% (Silva *et al.*, 2007). Yellow crystals suitable for X-ray analysis were grown from a solution of methanol and acetonitrile (1:1 *v*/*v*) at 298 K over a period of a few days in air.

Data collection: *APEX2* (Bruker, 2007); cell refinement: *APEX2* (Bruker, 2007); data reduction: *APEX2* (Bruker, 2007); Absorption correction: *SADABS* (Bruker, 2001); program used to solve structure: *SIR97* (Altomare *et al.*, 1999); program used to refinement structure: *SHELXL97* (Sheldrick, 2008); molecular graphics: *ORTEP-3* (Farrugia, 1997) and Mercury (Macrae *et al.*, 2006); software used to prepare material for publication: *WinGX* (Farrugia, 1999); geometric calculations: *PLATON* (Spek, 2003).

### Refinement

The H8 atom was located in a difference map and refined. The rest of the H atoms were positioned geometrically and refined with use of a riding model with C—H = 0.95 - 0.99Å and *U*_iso_ = 1.2 times *U*_eq_ of the bonded C. Friedel pairs were merged for the final refinement.

### Crystal data

**Table:**

C_12_H_12_N_4_O_3_	*F*_000_ = 272
*M**_r_* = 260.26	*D*_x_ = 1.446 Mg m^−^^3^
Monoclinic, *P*2_1_	Mo *K*α radiation λ = 0.7107 Å
Hall symbol: P 2yb	Cell parameters from 1992 reflections
*a* = 4.65560 (10) Å	θ = 3.0–28.3º
*b* = 10.9113 (2) Å	µ = 0.11 mm^−^^1^
*c* = 11.7681 (3) Å	*T* = 100 (2) K
β = 90.6680 (10)º	Prism, colourless
*V* = 597.76 (2) Å^3^	0.34 × 0.16 × 0.12 mm
*Z* = 2	

### Data collection

**Table:**

Bruker APEXII CCD diffractometer	1287 independent reflections
Radiation source: fine-focus sealed tube	1216 reflections with *I* > 2σ(*I*)
Monochromator: graphite	*R*_int_ = 0.012
*T* = 100(2) K	θ_max_ = 26.4º
ω and φ scans	θ_min_ = 1.7º
Absorption correction: multi-scan(SADABS; Bruker, 2001)	*h* = −5→5
*T*_min_ = 0.861, *T*_max_ = 0.987	*k* = −13→13
11597 measured reflections	*l* = −14→14

### Refinement

**Table:**

Refinement on *F*^2^	Secondary atom site location: difference Fourier map
Least-squares matrix: full	Hydrogen site location: inferred from neighbouring sites
*R*[*F*^2^ > 2σ(*F*^2^)] = 0.025	H atoms treated by a mixture of independent and constrained refinement
*wR*(*F*^2^) = 0.066	*w* = 1/[σ^2^(*F*_o_^2^) + (0.0385*P*)^2^ + 0.0782*P*] where *P* = (*F*_o_^2^ + 2*F*_c_^2^)/3
*S* = 1.08	(Δ/σ)_max_ = 0.001
1287 reflections	Δρ_max_ = 0.14 e Å^−^^3^
176 parameters	Δρ_min_ = −0.22 e Å^−^^3^
1 restraint	Extinction correction: none
Primary atom site location: structure-invariant direct methods	

### Special details

**Table:**

Geometry. All e.s.d.'s (except the e.s.d. in the dihedral angle between two l.s. planes) are estimated using the full covariance matrix. The cell e.s.d.'s are taken into account individually in the estimation of e.s.d.'s in distances, angles and torsion angles; correlations between e.s.d.'s in cell parameters are only used when they are defined by crystal symmetry. An approximate (isotropic) treatment of cell e.s.d.'s is used for estimating e.s.d.'s involving l.s. planes.
Refinement. Refinement of *F*^2^ against ALL reflections. The weighted *R*-factor *wR* and goodness of fit *S* are based on *F*^2^, conventional *R*-factors *R* are based on *F*, with *F* set to zero for negative *F*^2^. The threshold expression of *F*^2^ > σ(*F*^2^) is used only for calculating *R*-factors(gt) *etc*. and is not relevant to the choice of reflections for refinement. *R*-factors based on *F*^2^ are statistically about twice as large as those based on *F*, and *R*- factors based on ALL data will be even larger.

### Fractional atomic coordinates and isotropic or equivalent isotropic displacement parameters (Å^2^)

**Table:**

	*x*	*y*	*z*	*U*_iso_*/*U*_eq_	
C1	0.4003 (3)	0.40584 (12)	0.08674 (11)	0.0161 (3)	
C2	0.2787 (3)	0.42354 (13)	−0.02020 (12)	0.0185 (3)	
H2	0.3368	0.4915	−0.0649	0.022*	
C3	0.0725 (3)	0.34290 (15)	−0.06277 (12)	0.0228 (3)	
H3	−0.0096	0.3558	−0.1361	0.027*	
C4	−0.0126 (3)	0.24393 (15)	0.00205 (14)	0.0249 (3)	
H4	−0.1559	0.1896	−0.0263	0.03*	
C5	0.1101 (3)	0.22366 (13)	0.10809 (13)	0.0245 (3)	
H5	0.0536	0.1548	0.1518	0.029*	
C6	0.3162 (3)	0.30441 (13)	0.15048 (12)	0.0199 (3)	
H6	0.4003	0.2904	0.2233	0.024*	
C7	0.6208 (3)	0.49433 (14)	0.13271 (12)	0.0183 (3)	
H7A	0.7996	0.4494	0.1521	0.022*	
H7B	0.6664	0.5558	0.0737	0.022*	
N8	0.5145 (3)	0.55688 (11)	0.23428 (10)	0.0162 (3)	
H8	0.338 (4)	0.5619 (16)	0.2452 (14)	0.019 (4)*	
C9	0.6898 (3)	0.62199 (12)	0.30060 (11)	0.0136 (3)	
O10	0.94844 (19)	0.63427 (9)	0.28473 (8)	0.0174 (2)	
C11	0.5490 (3)	0.67480 (13)	0.40651 (12)	0.0169 (3)	
H11A	0.3579	0.7085	0.3859	0.02*	
H11B	0.5211	0.6087	0.4629	0.02*	
N12	0.7269 (2)	0.77163 (10)	0.45668 (9)	0.0155 (2)	
C13	0.7665 (3)	0.89028 (13)	0.42442 (11)	0.0173 (3)	
N14	0.9577 (3)	0.94933 (12)	0.48657 (10)	0.0212 (3)	
C15	1.0495 (3)	0.86385 (13)	0.56313 (12)	0.0202 (3)	
H15	1.1901	0.8781	0.6208	0.024*	
C16	0.9113 (3)	0.75456 (13)	0.54557 (11)	0.0178 (3)	
H16	0.939	0.681	0.5875	0.021*	
N17	0.6082 (3)	0.94740 (12)	0.33348 (10)	0.0239 (3)	
O18	0.4426 (2)	0.88404 (11)	0.27695 (9)	0.0306 (3)	
O19	0.6452 (3)	1.05789 (11)	0.31808 (11)	0.0419 (3)	

### Atomic displacement parameters (Å^2^)

**Table:**

	*U*^11^	*U*^22^	*U*^33^	*U*^12^	*U*^13^	*U*^23^
C1	0.0137 (6)	0.0156 (7)	0.0191 (6)	0.0026 (6)	0.0028 (5)	−0.0051 (5)
C2	0.0202 (7)	0.0166 (7)	0.0187 (7)	0.0025 (6)	0.0021 (5)	−0.0016 (5)
C3	0.0208 (7)	0.0276 (8)	0.0199 (7)	0.0040 (7)	−0.0040 (6)	−0.0089 (6)
C4	0.0191 (7)	0.0216 (8)	0.0340 (8)	−0.0045 (6)	0.0008 (6)	−0.0137 (6)
C5	0.0251 (8)	0.0152 (8)	0.0333 (8)	−0.0025 (6)	0.0055 (6)	−0.0029 (6)
C6	0.0212 (7)	0.0192 (8)	0.0193 (7)	−0.0006 (6)	0.0004 (5)	−0.0023 (6)
C7	0.0161 (7)	0.0196 (7)	0.0193 (7)	−0.0006 (6)	0.0030 (5)	−0.0047 (6)
N8	0.0106 (6)	0.0182 (6)	0.0198 (6)	−0.0003 (5)	0.0016 (4)	−0.0057 (5)
C9	0.0153 (7)	0.0096 (6)	0.0159 (6)	0.0013 (5)	0.0000 (5)	0.0008 (5)
O10	0.0134 (5)	0.0189 (5)	0.0198 (4)	−0.0008 (4)	0.0010 (4)	−0.0031 (4)
C11	0.0160 (6)	0.0155 (7)	0.0194 (7)	−0.0036 (6)	0.0012 (5)	−0.0059 (5)
N12	0.0168 (6)	0.0139 (6)	0.0159 (5)	−0.0011 (5)	0.0020 (4)	−0.0024 (5)
C13	0.0221 (7)	0.0133 (7)	0.0164 (6)	0.0005 (6)	−0.0001 (5)	−0.0013 (5)
N14	0.0281 (7)	0.0169 (6)	0.0187 (6)	−0.0036 (6)	−0.0021 (5)	−0.0029 (5)
C15	0.0236 (8)	0.0176 (7)	0.0194 (7)	0.0005 (6)	−0.0037 (6)	−0.0044 (5)
C16	0.0210 (7)	0.0153 (7)	0.0171 (6)	0.0029 (6)	−0.0014 (5)	−0.0014 (5)
N17	0.0325 (8)	0.0211 (7)	0.0179 (6)	0.0008 (6)	−0.0037 (5)	0.0002 (5)
O18	0.0347 (6)	0.0323 (7)	0.0244 (5)	−0.0023 (5)	−0.0114 (5)	−0.0012 (5)
O19	0.0699 (10)	0.0196 (6)	0.0358 (6)	−0.0031 (6)	−0.0180 (6)	0.0077 (5)

### Geometric parameters (Å, °)

**Table:**

O10—C9	1.2281 (16)	C15—C16	1.370 (2)
N17—O18	1.2260 (17)	C15—H15	0.95
N17—O19	1.2316 (18)	C4—C3	1.383 (2)
N17—C13	1.4348 (18)	C4—C5	1.384 (2)
N12—C16	1.3581 (18)	C4—H4	0.95
N12—C13	1.3622 (19)	C5—C6	1.391 (2)
N12—C11	1.4628 (17)	C5—H5	0.95
N8—C9	1.3286 (17)	C3—H3	0.95
N8—C7	1.4675 (17)	C7—C1	1.505 (2)
N8—H8	0.836 (18)	C7—H7A	0.99
C9—C11	1.5278 (18)	C7—H7B	0.99
C2—C1	1.3875 (19)	C1—C6	1.396 (2)
C2—C3	1.391 (2)	C11—H11A	0.99
C2—H2	0.95	C11—H11B	0.99
N14—C13	1.3144 (19)	C16—H16	0.95
N14—C15	1.3620 (19)	C6—H6	0.95
			
O18—N17—O19	124.07 (13)	C4—C3—H3	120.1
O18—N17—C13	118.30 (12)	C2—C3—H3	120.1
O19—N17—C13	117.62 (13)	N8—C7—C1	110.86 (11)
C16—N12—C13	104.99 (12)	N8—C7—H7A	109.5
C16—N12—C11	124.17 (12)	C1—C7—H7A	109.5
C13—N12—C11	130.67 (12)	N8—C7—H7B	109.5
C9—N8—C7	121.09 (12)	C1—C7—H7B	109.5
C9—N8—H8	118.2 (12)	H7A—C7—H7B	108.1
C7—N8—H8	119.9 (11)	C2—C1—C6	118.93 (13)
O10—C9—N8	124.46 (12)	C2—C1—C7	120.41 (13)
O10—C9—C11	120.91 (12)	C6—C1—C7	120.65 (12)
N8—C9—C11	114.48 (11)	N12—C11—C9	110.81 (11)
C1—C2—C3	120.75 (13)	N12—C11—H11A	109.5
C1—C2—H2	119.6	C9—C11—H11A	109.5
C3—C2—H2	119.6	N12—C11—H11B	109.5
C13—N14—C15	103.74 (12)	C9—C11—H11B	109.5
N14—C15—C16	110.69 (12)	H11A—C11—H11B	108.1
N14—C15—H15	124.7	N12—C16—C15	106.76 (13)
C16—C15—H15	124.7	N12—C16—H16	126.6
C3—C4—C5	120.29 (14)	C15—C16—H16	126.6
C3—C4—H4	119.9	C5—C6—C1	120.41 (13)
C5—C4—H4	119.9	C5—C6—H6	119.8
C4—C5—C6	119.86 (14)	C1—C6—H6	119.8
C4—C5—H5	120.1	N14—C13—N12	113.81 (12)
C6—C5—H5	120.1	N14—C13—N17	122.74 (13)
C4—C3—C2	119.74 (13)	N12—C13—N17	123.42 (12)
			
C7—N8—C9—O10	−1.0 (2)	C11—N12—C16—C15	176.45 (12)
C7—N8—C9—C11	−176.53 (13)	N14—C15—C16—N12	−0.46 (16)
C13—N14—C15—C16	0.00 (16)	C4—C5—C6—C1	0.0 (2)
C3—C4—C5—C6	1.1 (2)	C2—C1—C6—C5	−1.1 (2)
C5—C4—C3—C2	−1.1 (2)	C7—C1—C6—C5	179.30 (13)
C1—C2—C3—C4	0.0 (2)	C15—N14—C13—N12	0.48 (16)
C9—N8—C7—C1	167.75 (12)	C15—N14—C13—N17	178.56 (13)
C3—C2—C1—C6	1.1 (2)	C16—N12—C13—N14	−0.77 (16)
C3—C2—C1—C7	−179.29 (13)	C11—N12—C13—N14	−176.13 (13)
N8—C7—C1—C2	116.20 (14)	C16—N12—C13—N17	−178.83 (13)
N8—C7—C1—C6	−64.20 (17)	C11—N12—C13—N17	5.8 (2)
C16—N12—C11—C9	−97.72 (15)	O18—N17—C13—N14	177.06 (14)
C13—N12—C11—C9	76.86 (17)	O19—N17—C13—N14	−3.8 (2)
O10—C9—C11—N12	20.40 (18)	O18—N17—C13—N12	−5.0 (2)
N8—C9—C11—N12	−163.87 (11)	O19—N17—C13—N12	174.08 (14)
C13—N12—C16—C15	0.70 (14)		

### Hydrogen-bond geometry (Å, °)

**Table:**

*D*—H···*A*	*D*—H	H···*A*	*D*···*A*	*D*—H···*A*
N8—H8···O10^i^	0.835 (19)	2.037 (18)	2.837 (2)	160.2 (17)

Symmetry codes: (i) *x*−1, *y*, *z*.
